# Clinical Symptoms of Skin, Nails, and Joints Manifest Independently in Patients with Concomitant Psoriasis and Psoriatic Arthritis

**DOI:** 10.1371/journal.pone.0020279

**Published:** 2011-06-01

**Authors:** Knut M. Wittkowski, Craig Leonardi, Alice Gottlieb, Alan Menter, Gerald G. Krueger, Paul W. Tebbey, Jennifer Belasco, Razieh Soltani-Arabshahi, John Gray, Liz Horn, James G. Krueger, for the International Psoriasis Council

**Affiliations:** 1 The Rockefeller University, New York, New York, United States of America; 2 St. Louis University Medical School, St. Louis University, St. Louis, Missouri, United States of America; 3 Tufts Medical Center, Tufts University, Boston, Massachusetts, United States of America; 4 Baylor Institute of Research, Dermatology Research Center, Baylor University Medical Center, Baylor University, Dallas, Texas, United States of America; 5 University of Utah, Salt Lake City, Utah, United States of America; 6 International Psoriasis Council, Dallas, Texas, United States of America; Harvard Medical School; Beth Israel, United States of America

## Abstract

This study correlated assessment tools for evaluating the severity of skin, nail, and joint symptoms in patients with psoriasis (Pso) and psoriatic arthritis (PsA). Adults with plaque Pso (with or without PsA) were enrolled from four U.S. institutions. Patients were evaluated using a novel 10-area Linear Psoriasis Area and Severity Index (XL-PASI), Psoriatic Arthritis Assessment (PsAA), Psoriatic Arthritis Screening and Evaluation Questionnaire (PASE), Nail Assessment (NA) and Joint Assessment (JA) tools, Psoriasis Weighted Extent and Severity Index (PWESI), and Lattice Physician Global Assessment (LS-PGA). Correlations between assessment tools and individual items in the assessment tools were performed. Data from 180 patients (55 with PsA) were analyzed. Highest correlations between tools (r = 0.77–0.88) were between the XL-PASI, PWESI and LS-PGA. Individual items in the XL-PASI correlated with items in the PWESI for extent skin symptoms, but not for all body areas. Overall, correlations were seen between hands and feet, between face and scalp, and between buttocks, chest, and back. Only low correlation was seen between items assessing joint symptoms with items assessing skin symptoms. These data support the notion that the complex phenotype of psoriatic disease requires instruments that assess the severity of skin, nails, and joints separately.

## Introduction

Psoriasis (Pso) is a chronic, systemic, inflammatory, multigenic disease, that manifests in the skin and has significant impact on quality of life [1]. About one quarter of patients with Pso also develop psoriatic arthritis (PsA) [2]. Nail involvement is common in patients with Pso and more so with PsA [3]. Recent studies have suggested that psoriatic skin, joint, and nail manifestations, long believed to share a common etiology, may be traits of distinct disease conditions [4]–[8]. This may explain why the severity and progression of skin, joint, and nail symptoms are frequently asynchronous [9], with joint disease in most patients developing up to 10 years after the initial skin presentation.

Genes within the Major Histocompatibility Complex and genes encoding cytokines have been linked to Pso and PsA [1], [10]–[14]. Genetic evidence supports the notion that Pso and PsA are two disease conditions with comorbid association, but might have distinct genes that drive pathogenesis, progression, and severity [1], [10], [15]. Individual alleles for many of these genes and single nucleotide polymorphisms (SNPs) within these genes appear to be differentially associated with Pso and PsA. Recently, Nograles et al. postulated that Pso and PsA involve multiple genes with low risk effects, only some of which may be shared [16].

Biological evidence also supports a physiological differentiation. For example, selected key immune mediators of disease in the skin and joints differ. Pathologic events in the skin are mediated by autoreactive T cells [1], whereas T cells in the affected synovium of PsA patients do not exhibit the same type of autoreactivity [17]. Skin and joint symptoms frequently do not respond equally or in parallel to systemic or biologic therapeutic agents [18].

Hence, there is a need to optimize the diagnosis and recording of the appearance and progression of Pso and PsA for both clinical research purposes and clinical practice, thus optimizing treatment modalities and outcomes. The goal of this paper is to determine from existing tools the smallest number of independent scoring dimensions needed to measure the presence and severity of skin, nail and joint involvement in the broadest psoriatic patient population. Various assessment tools were then evaluated in a phase II-type clinical trial.

## Methods

### Study design

The clinical portion of the study was conducted from November 2007 to August 2008 in four U.S. institutions; Baylor Research Institute, Dallas; Central Dermatology, St. Louis; University of Utah, Salt Lake City; and Tufts Medical Center, Boston. The study protocol and informed consent forms were approved by all institutional review boards and all patients signed a written consent form before initiation of study-specific procedures. All statistical analyses were performed at the Rockefeller University, Center for Clinical and translational Science, New York.

### Patients

Eligible patients were >18 years old and had plaque Pso (with or without PsA) with ≥5% and ≤90% body surface area (BSA) affected.

### Disease assessment tools

As described below, the assessment tools comprised two categories: those that involved a physician examination and those submitted to the patient by questionnaire. Some tools were modified slightly to derive more detailed information and consequently, these are included as supplemental figures.

#### Psoriasis Area and Severity Index (PASI)

The PASI is the most commonly used tool to assess disease severity in patients with Pso in clinical trials [19]. The PASI measures erythema, scaling, and thickness of lesions and is weighted by the area of involvement. The PASI scale ranges from 0 to 72, calculated as the extent of involvement × (score for erythema + score for scaling + score for thickness), where the extent of involvement is categorized as: 0 (0%), 1 (1–9%), 2 (10–29%), 3 (30–49%), 4 (50–69%), 5 (70–89%), or 6 (90–100%), and scores for erythema, scaling, and thickness are each rated on a scale of 0 to 4. It is seldom used in clinical practice.

#### Psoriatic Arthritis Assessment (PsAA)

The PsAA (Fig. S1) is a composite measurement of PsA that includes questions regarding patient and family history and 6 multipart components of a physical examination. *Psoriatic Arthritis Screening and Evaluation Questionnaire (PASE)*. This validated questionnaire (Fig. S2) comprises seven questions regarding symptoms and eight questions regarding function that are answered by the patient [20]. Questions are rated on a scale of 1 (strongly disagree) to 5 (strongly agree).

#### Nail Assessment (NA) and Joint Assessment (JA)

The NA and JA (Fig. S3) evaluate nail involvement (score 0 for no involvement and score 1 for involvement by digit) and joint involvement (0/1 by digit) on the hands. The scoring is based upon physician assessment. Selected items that evaluate the type of disease for most involved nail were excluded as several assessors had included toenails; only fingernail assessments were included in this analysis.

#### Psoriasis Weighted Extent and Severity Index (PWESI)

This index (Fig. S4) is used to evaluate the extent of skin disease on a scale of 0 (none) to 4 (extensive) and severity of disease on a scale of 0 (essentially clear) to (intensely inflamed) (46). Ten areas are assessed: scalp/hairline, face/neck, arms/axillae, hands/fingers/fingernails, anterior chest/abdomen, back/shoulders, genitalia/perineum/natal cleft, buttocks/thighs, knees/lower legs/ankles, and feet/toes/toenails. The physician assessment delivers a maximal composite score of 50.

#### Extended 10-Area Linear Psoriasis Area and Severity Index (XL-PASI)

The XL-PASI (illustrated in Fig. S5) is novel assessment tool combines the PASI method [19] of scoring severity separately by erythema, thickness, and scaling with the PWESI method of dividing the body surface into ten areas. The modified PASI measure includes assessment of surface area involved, as well as dimensions for scaling, erythema, thickness and joint involvement for specific areas of psoriatic involvement. As with the PASI, severity indicators range from 0 (none) to 4 (extremely severe). As with the PWESI, body surface area was divided into ten (“X”) areas. To increase the granularity of the results, each area was quantified (“Linearized”) rather than categorized, i.e., the proportion of area affected was estimated numerically, rather than merely broadly categorized as in the PASI. Finally, the PASI was “eXtended” to include severity indicators for arthritis, quality of life, and sensations (pain/itching), although these additional measures were not included in this study. The scale of the XL-PASI ranges from 0 to 148.

#### Lattice Physician Global Assessment (LS-PGA)

The LS-PGA [21] was included in the analysis of correlations between assessment tools. The results were deemed not sufficiently detailed to be included in the itemized analysis.

### Data entry

Electronic versions of the instruments were generated using the Web-based Interactive Study Design, Organization, and Management (WISDOM) software, and are available for download at http://wisdom.rockefeller.edu. The meta data were extracted and automatically reformatted as screen form definitions for Research Electronic Data Capturing (REDCap) [22]. Data were entered twice by different typists and inconsistencies were resolved by an independent reviewer. One assessor entered the percentage total body area involvement as the XL-PASI proportion of involved skin, rather than percentage of body area by region. These data were analyzed as interval-censored between the highest and lowest value consistent with the input [23].

### Statistical analyses

Statistical analyses were conducted using the μStat server at the Rockefeller University (http://mustat.rockefeller.edu) for computing u-scores of the binary, discrete, continuous, and interval-censored data. JMP® 7.0 software (The SAS Institute, Cary, NC) was used to generate the heatmap of the scores and Pearson correlations, equivalent to Spearman rank correlations [24]. R software (R Foundation, Vienna, Austria; http://cran.r-project.org) was used to compute odds ratios.

## Results

### Patients

Two hundred patients were screened for participation from the four clinical centers. Data from 180 evaluable patients that met the screening criteria were included in the analysis (Table 1). Of these, 55 (31%) patients had a diagnosis of PsA (confirmed by a rheumatologist) at baseline. The majority of patients were Caucasian (n = 154; 86%) and the remaining Asian (n = 5; 3%), Black (n = 6; 3%), Hispanic (n = 13; 7%), and Native American (n = 2; 1%).

**Table 1:** Patient demographics at baseline.

	Psoriasis^1^n = 182	PsA^1^n = 68	Nail Disease^1^n = 112
Sex (Female/Male)	84/98	32/36	43/69
Mean age - years (SD)Minimum, maximum	45.3 (15.0)18, 84	47.8 (13.6)20, 72	47.8 (13.8)20, 84
Mean weight - kg (SD)Minimum, maximum	204.9 (52.3)97, 377	207.5 (52.7)97, 350	209.1 (51.2)97, 377
Mean height - in (SD)Minimum, maximum	67.4 (4.3)50, 78	67.5 (3.8)59, 76	67.6 (4.2)50, 76
Patients with PsA diagnosis by a rheumatologist – n (%)	55 (30%)	53 (78%)	40 (36%)

1 Self-reported.

Abbreviations: PsA, Psoriatic arthritis; SD, Standard deviation.

### Correlations between assessment tools

**Figure 1:**
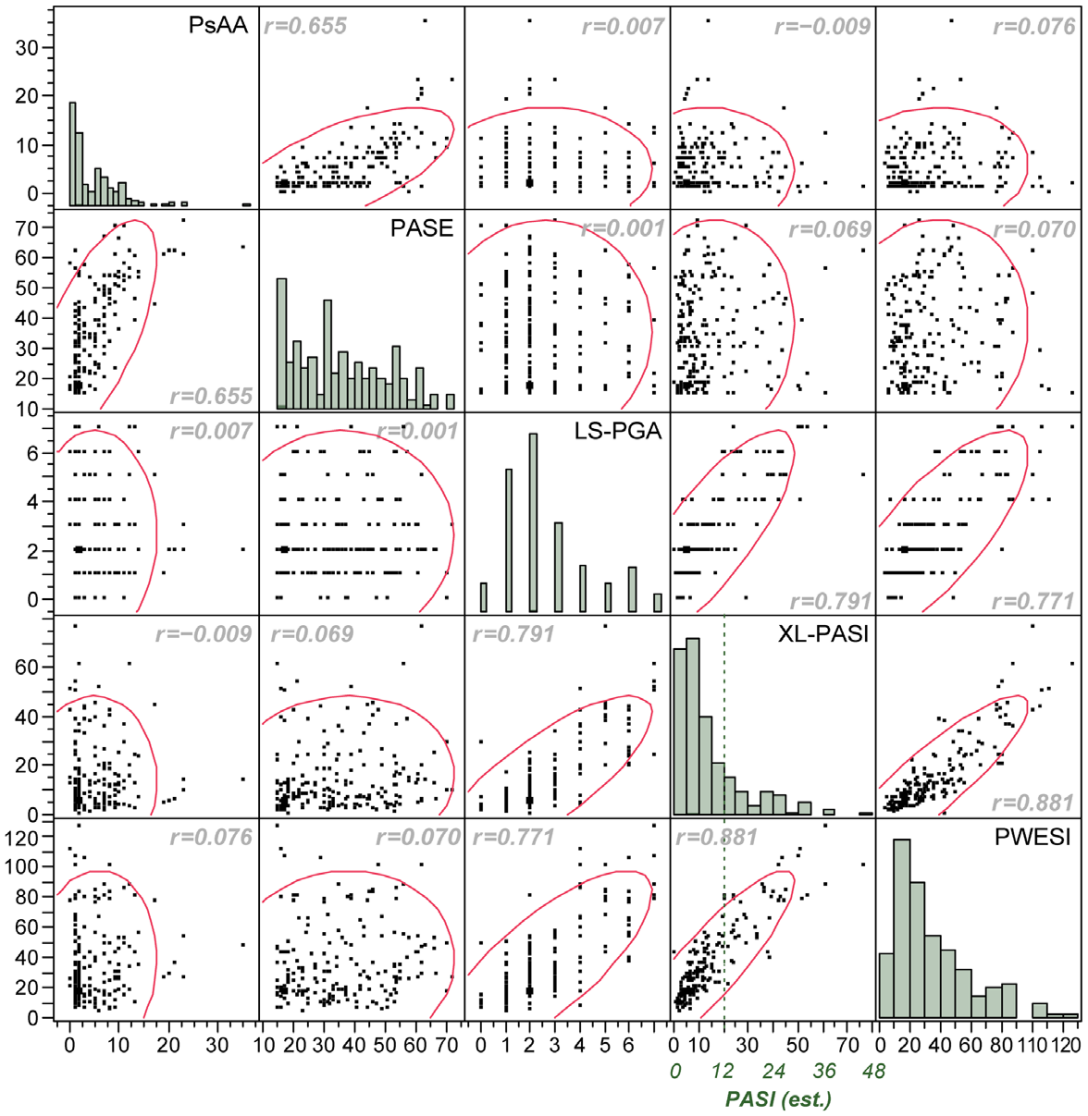
Correlations between tools scoring the severity of psoriasis and psoriatic arthritis and distribution of scores. x-axis and y-axis values represent the respective scores of the tools. For comparison with traditional PASI scores, the XL-PASI scores are translated into estimated PASI scores. The dotted line with an estimated PASI score of 12 separates the patients with moderate and severe disease. r: Spearman correlation.

Correlations between scoring tools used to assess severity of Pso and PsA are shown in Figure 1. The highest correlation was seen between the XL-PASI and the PWESI (r = 0.88), which differ in granularity and relative weight of their components. The LS-PGA was also reasonably well correlated with the XL-PASI and PWESI (r = 0.79 and 0.77, respectively). The PsAA and the PASE also demonstrated good correlation (r = 0.655), although the self-assessment tool (PASE) discriminated less between mild and severe cases. The subsequent detailed analysis showed a surprising absence of any correlation between these two groups of tools (PsAA/PASE) and LS-PGA/XL-PASI/PWESI.

### Correlations between items in the assessment tools

**Figure 2:**
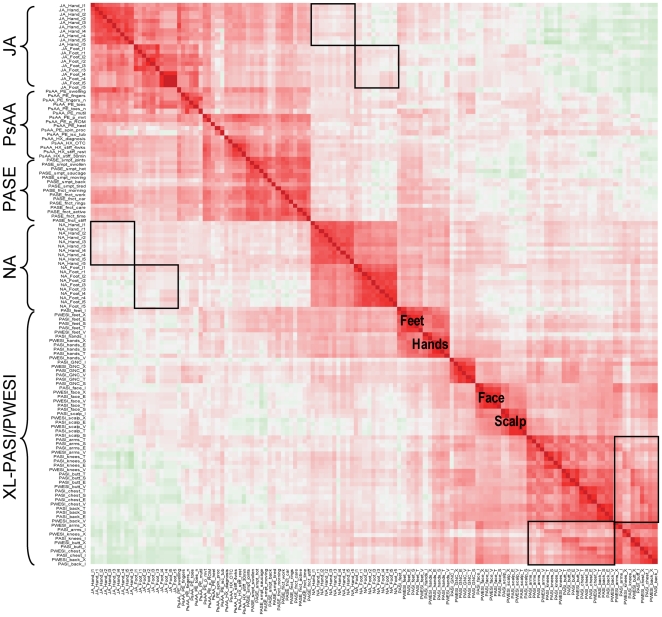
Correlations between individual items of psoriasis and psoriatic arthritis severity. Each cell indicates the Spearman correlation between 2 of the 52 items. Cells with dark red indicates strong positive correlation, light red indicates weak positive correlation, dark green indicates strong negative correlation, and light green indicates weak negative correlation. Boxes in the upper left portion indicate correlations between affected nails and joints on hands and feet. Boxes in the lower right corner indicate “secondary structure” (within-box diagonal) between extent/involvement and severity/activity within regions. JA: Joint Assessment; NA: Nail Assessment; PASE: Psoriatic Arthritis Screening and Evaluation Questionnaire; PsAA: Psoriatic Arthritis Assessment; XL-PASI: Extended 10-area Linear Psoriasis Area and Severity Index; PWESI: Psoriasis Weighted Extent and Severity Index.

Correlations between individual items in the scoring tools are shown in Figure 2. The observations among fingers (or toes) were highly correlated as indicated for joint involvement (upper left corner) and nail disease (center). Individual items in the PASI score and the corresponding PWESI extent score were also highly correlated, as were the three PASI disease activity scores (erythema, thickness, and scaling) and the PWESI severity score. The extent (surface area involved per PASI) and severity (activity per PASI) of disease were highly correlated for feet, hands, genitalia/perineum/natal cleft, face, and scalp. In contrast, severity/activity of arms, knees, buttocks, chest, and back did not correlate with the extent/involvement across these body areas. The “secondary structure” of off-diagonal correlations (between severity/activity and extent/involvement within the same region) in the lower right corner of Figure 2 indicated that these items were not independent (see Figure 2 legend for details). Overall, correlations were seen between hands and feet, face and scalp, and buttocks, chest, and back.

Little correlation was seen between the PASE, PsAA, and JA items describing severity of psoriatic arthritis and the PASI, PWESI, and NA. The only overlap noted among these items was individual PASE function items and extent and severity of Pso of the hands and feet. The correlations between hands/feet (XL-PASI/PWESI) and NA are likely inflated at the expense of the correlation between hands/feet and Pso of the other body areas (lower right corner) because of the design and wording of the questionnaires. Moreover, the NA items were highly correlated among each other, but had poor correlation with XL-PASI and PWESI items. Again, hands and feet were an exception, most notably for the severity by PWESI, which explicitly includes “fingernails” and “toenails” in its “hands” and “feet” categories. In particular, there were no secondary structures indicating that the joint and nail symptoms affect the same digits, an observation in contrast to the commonly believed theory.

## Discussion

The results of this study do not support the hypothesis that a “least common denominator” of individual components will suffice to define the severity of the spectrum of disease along the axes of both psoriasis and psoriatic arthritis. Thus, at this time, the need for a single measurement tool that is broadly utilizable with the ability to measure the presence and severity of all clinical aspects of psoriasis remains unmet.

Our results provide evidence against an association between the severity of joint and skin involvement in patients with Pso and PsA, with the possible exception of hand symptoms. There have been few studies that have directly addressed the controversial relationship between the complexities of skin, nail and joint disease in psoriatic patients. Several small studies have reported no [25]–[27] or weak [9] associations between the activity of Pso and the activity, severity or functional status of the joints. However, a correlation between skin and joint severity was noted in a small group of patients who had synchronous onset of PsA and Pso [27]. All of these studies had only small sample sizes and thus low statistical power. However, in a large phase 4 clinical trial of 1122 patients, Gottlieb et al., found that baseline skin and joint disease measures were not correlated [28]. While confirming these earlier findings, the present study is limited in regard to the statistical power describing the relationship of specific psoriatic arthritis subtypes or extent of psoriatic disease. Therefore, based upon these initial unexpected observations, a more robust analysis would be necessary to better investigate the skin-joint relationship.

However, in support of previous observations regarding a qualitative association between skin and joint disease [2], and consistent with both disease conditions sharing at least some genetic risk factors, we did find a high odds ratio (19.9; *P*<0.001) for skin involvement and “any joints involved” and “any nail involved” among toes and a moderate odds ratios (2.0; P = 0.079) for fingers. The lack of a quantitative association requires further investigation but might involve additional genetic risk factors.

Several inconsistencies in the design of individual instruments may have affected our results. The PWESI explicitly includes nails with hands and feet, but the guidelines provided on the form do not allow even the most severe nail disease to be marked more than “noticeable” in terms of extent. The XL-PASI (like the PASI), in contrast, does not mention nails at all. The diversity of Pso manifestations on the hands and feet (palmarplantar disease) for example hyperkeratosis vs pustular or combinations thereof, further complicates the issue (Farley et al., 2009). The relatively high correlation between severity of disease and extent of disease in nails and hands/feet and the extremely low correlation between extent of disease in hands/feet and extent of disease in all other body areas may suggest that the examiners attempted to reflect severe nail disease by inflating extent and severity of skin disease of hands and feet. The XL-PASI includes a column for severity of arthritis, so that physicians were less likely to reflect joint problems by increasing the severity indicators meant to reflect skin conditions. The PWESI, by contrast, has no separate category for arthritis and, in support of this argument, had a higher correlation with severity of arthritis than the PASI.

Analysis of data to evaluate nail disease was complicated by wording of the assessment tools. The NA includes a request for a list of all involved digits (fingers and toes), but then continues with “Select the most involved fingernail and evaluate.” Several completed questionnaires indicated that the examiner had included toenails in the assessment, but others may have followed the instructions literally so that the assessment could be biased to disease forms more frequently seen with hands, such as pitting. This issue highlights the importance of validating assessment tools in the appropriate clinical setting. These observations suggest that Pso of the nails might be more clearly separated from Pso of the skin if examiners were allowed to distinguish explicitly between involvement of nails and skin on both hands and feet.

The pathophysiology of nail dystrophy has been postulated to be more closely associated with joint symptoms than with skin symptoms [4]. As with the hands and feet, there is a great diversity in the clinical manifestations of nail disease, with the added problem of secondary infections (bacterial, fungal) aggravating or masking the manifestations, particularly in psoriatic toenails. PsA has been more strongly associated with nail disease than Pso alone [29]. Imaging studies have shown that the nail and the enthesis (the point at which tendons attach to bones) are linked via the distal interphalangeal joint extensor tendon [30], [31]. This close functional relationship between the joint and the nail may explain the association between these symptoms, particularly relating to distal interphalangeal joint involvement and nail disease.

GWAS and family-based association tests for Pso as a whole have had only limited success in defining causative alleles. The results presented here, however, offer a strategy for future genetic studies with a high potential for success in elucidating genetic risk factors for Pso. More specific and statistically validated assessment instruments will allow for the clinical classification of Pso patients into specific categories based on skin, joint, and nail involvement. In addition, the identification of genetic discriminators between these categories, using novel multivariate [23], multi-locus [32], and multi-allelic [33] nonparametric methods in a well-defined subpopulation of patients with the same underlying condition, would be more accurate than studies using traditional methods for univariate, single-locus, or binary data in the more diverse population as a whole.

The clinical evidence presented herein likely signifies the existence of different psoriatic disease phenotypes, thus supporting the hypothesis that different genetic risk factors are involved in the etiology of skin, joint, and possibly nail forms of psoriatic disease. Our results suggest that the complex phenotype of psoriatic disease can only be assessed with instruments that allow the examiner to judge severity of skin, nails, and joints separately (in addition to extent of skin involvement). Moreover, the data analysis suggests that a minimum of two summary scores (one for skin and one for joints), and potentially a third for nails, are required to accurately assess severity across the full spectrum of psoriatic disease. The optimal design of such assessment tools remains the objective of this research project, with efforts continuing to identify the most meaningful contributing elements that define the full spectrum of the psoriatic disease state. Accordingly, this information is likely to be of significant benefit clinical practice as well as in the future design of clinical trials investigating developmental therapies for Pso and PsA.

## Supporting Information

Figure S1 **Psoriatic Arthritis Assessment (PsAA) is a composite measurement of PsA that includes questions regarding patient and family history and 6 multipart components of a physical examination.**
(TIF)

Figure S2 **Psoriatic Arthritis Screening and Evaluation (PASE) is a validated patient questionnaire that comprises questions on symptoms and functions.**
(TIF)

Figure S3 **Nail Assessment (NA) and Joint Assessment (JA) evaluates nail involvement (score 0 for no involvement and score 1 for involvement by digit) and joint involvement (0/1 by digit) on the hands, as determined by physician assessment.**
(TIF)

Figure S4 **Psoriasis Weighted Extent and Severity Index (PWESI) is used to evaluate the extent and severity of skin disease across multiple areas of the body.** The extent of skin disease is measured on a scale of 0 (none) to 4 (extensive) and severity of disease on a scale of 0 (essentially clear) to (intensely inflamed). Ten areas are assessed: scalp/hairline, face/neck, arms/axillae, hands/fingers/fingernails, anterior chest/abdomen, back/shoulders, genitalia/perineum/natal cleft, buttocks/thighs, knees/lower legs/ankles, and feet/toes/toenails. This physician assessment delivers a maximal composite score of 50.
(TIF)

Figure S5 **Extended 10-area Linear Psoriasis Area and Severity Index (XL-PASI) combines the PASI and PWESI scoring methods.** This measure includes assessment of surface area involved, as well as dimensions for scaling, erythema, thickness and joint involvement for specific areas of psoriatic involvement. As with the PASI, severity indicators range from 0 (none) to 4 (extremely severe). As with the PWESI, body surface area is divided into ten (“X”) areas and each area is quantified. The scale of the XL-PASI ranges from 0 to 148.
(TIF)
