# Supplementary figures and images for: Clinical Symptoms of Skin, Nails, and Joints Manifest Independently in Patients with Concomitant Psoriasis and Psoriatic Arthritis
Source: PLoS One. 2011 Jun 1;6(6):e20279. doi: 10.1371/journal.pone.0020279 (PMC3106005; doi:10.1371/journal.pone.0020279)

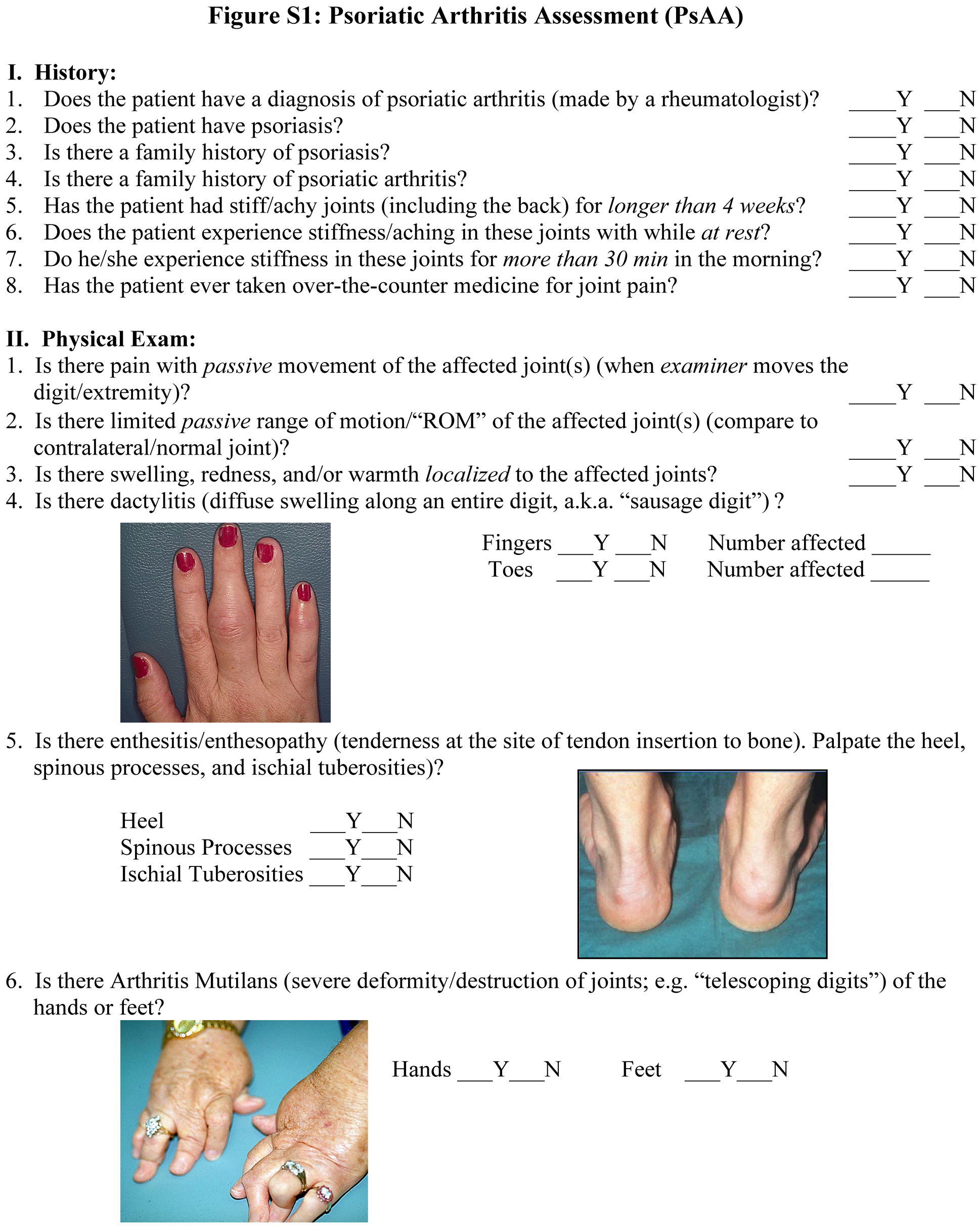

Supplement: Figure S1 — Psoriatic Arthritis Assessment (PsAA) is a composite measurement of PsA that includes questions regarding patient and family history and 6 multipart components of a physical examination. (TIF) [file pone.0020279.s001.tif]

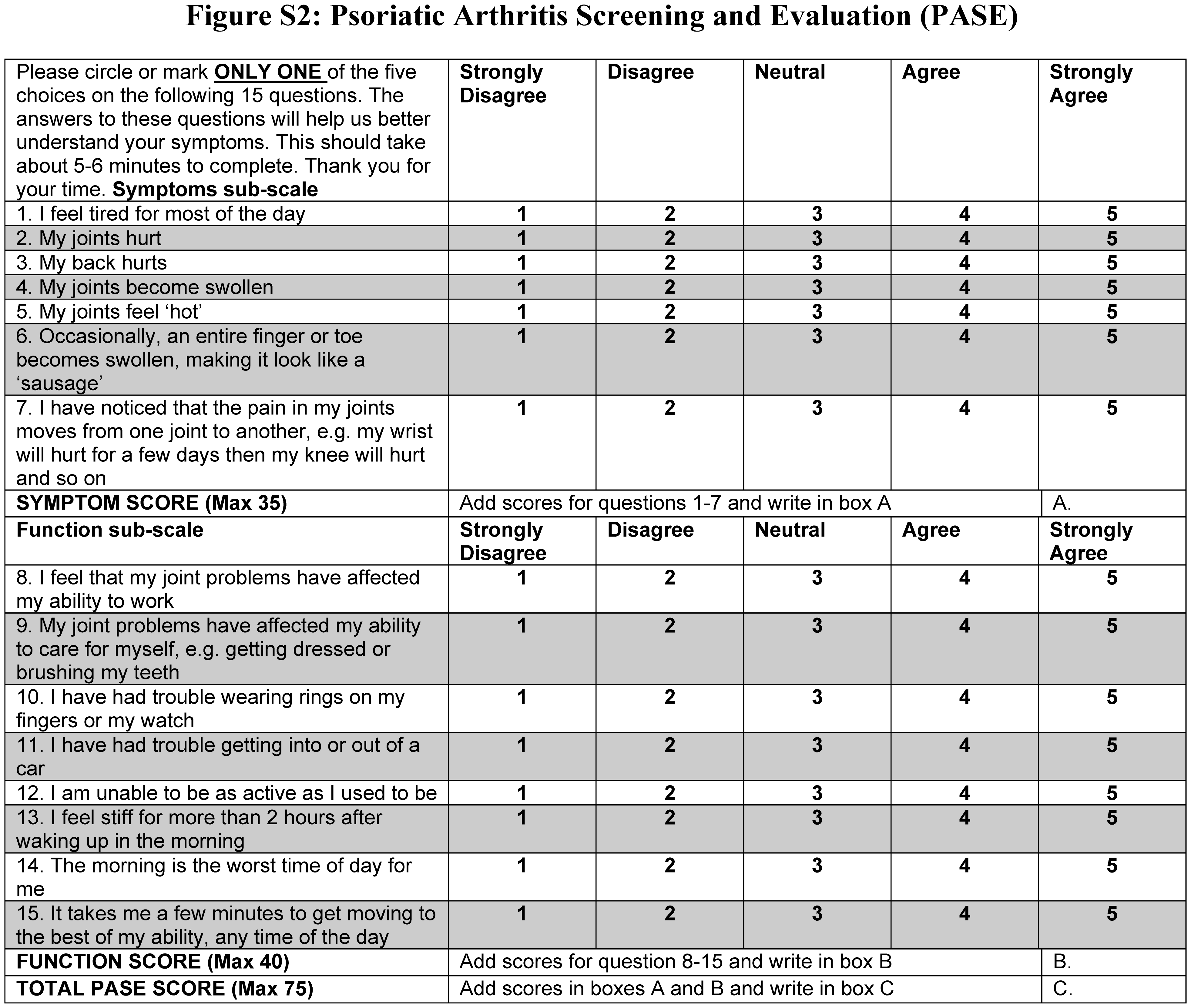

Supplement: Figure S2 — Psoriatic Arthritis Screening and Evaluation (PASE) is a validated patient questionnaire that comprises questions on symptoms and functions. (TIF) [file pone.0020279.s002.tif]

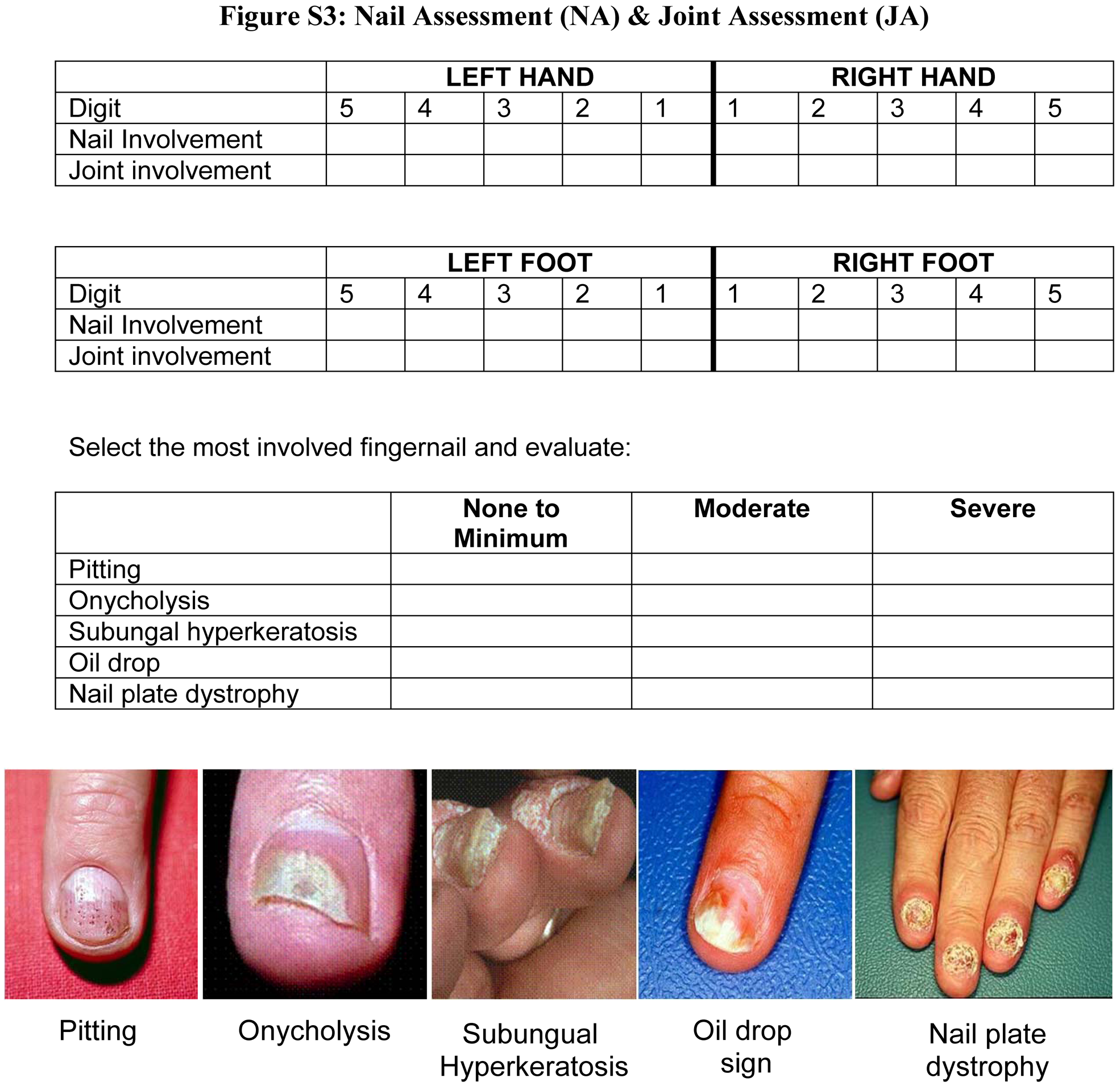

Supplement: Figure S3 — Nail Assessment (NA) and Joint Assessment (JA) evaluates nail involvement (score 0 for no involvement and score 1 for involvement by digit) and joint involvement (0/1 by digit) on the hands, as determined by physician assessment. (TIF) [file pone.0020279.s003.tif]

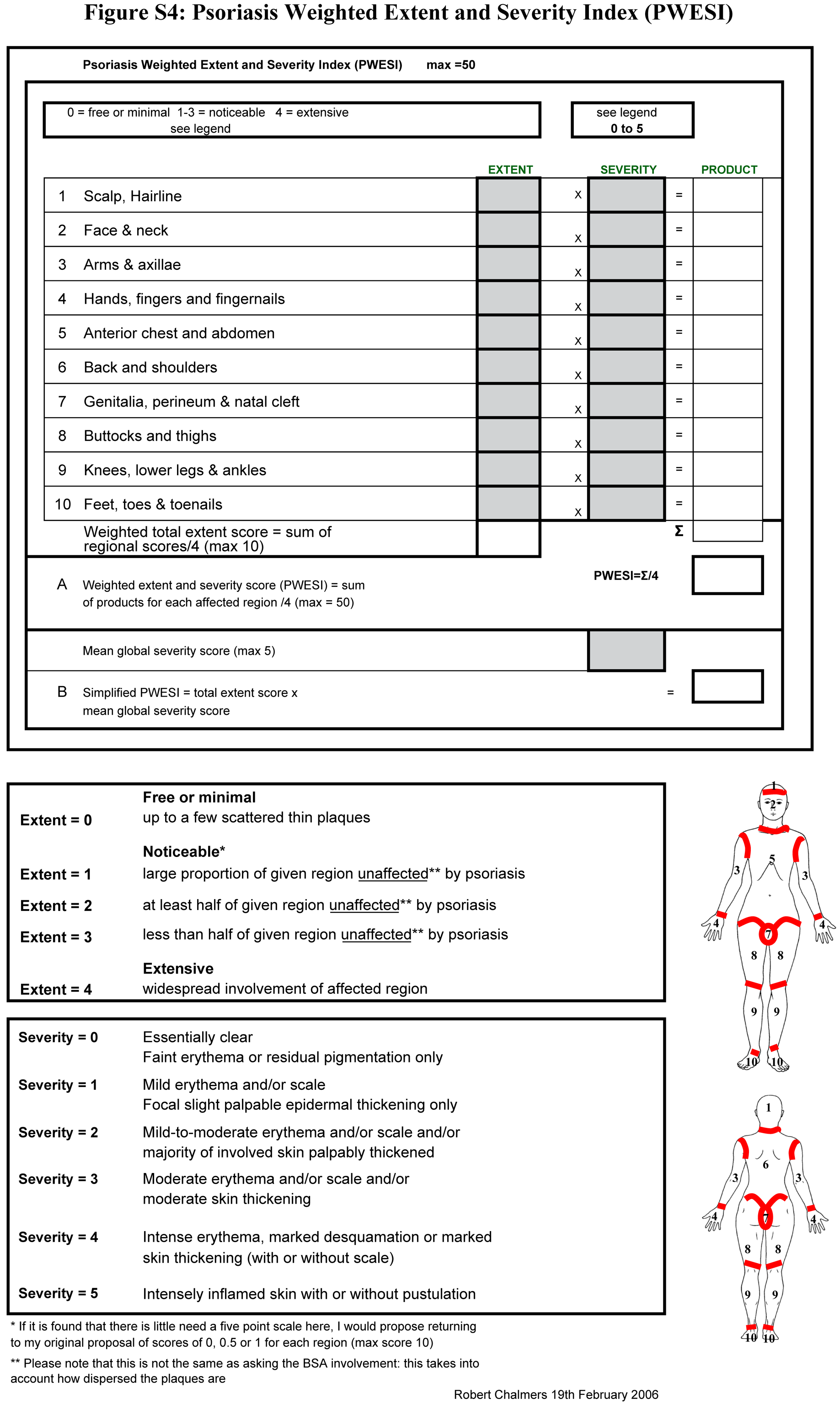

Supplement: Figure S4 — Psoriasis Weighted Extent and Severity Index (PWESI) is used to evaluate the extent and severity of skin disease across multiple areas of the body. The extent of skin disease is measured on a scale of 0 (none) to 4 (extensive) and severity of disease on a scale of 0 (essentially clear) to (intensely inflamed). Ten areas are assessed: scalp/hairline, face/neck, arms/axillae, hands/fingers/fingernails, anterior chest/abdomen, back/shoulders, genitalia/perineum/natal cleft, buttocks/thighs, knees/lower legs/ankles, and feet/toes/toenails. This physician assessment delivers a maximal composite score of 50. (TIF) [file pone.0020279.s004.tif]

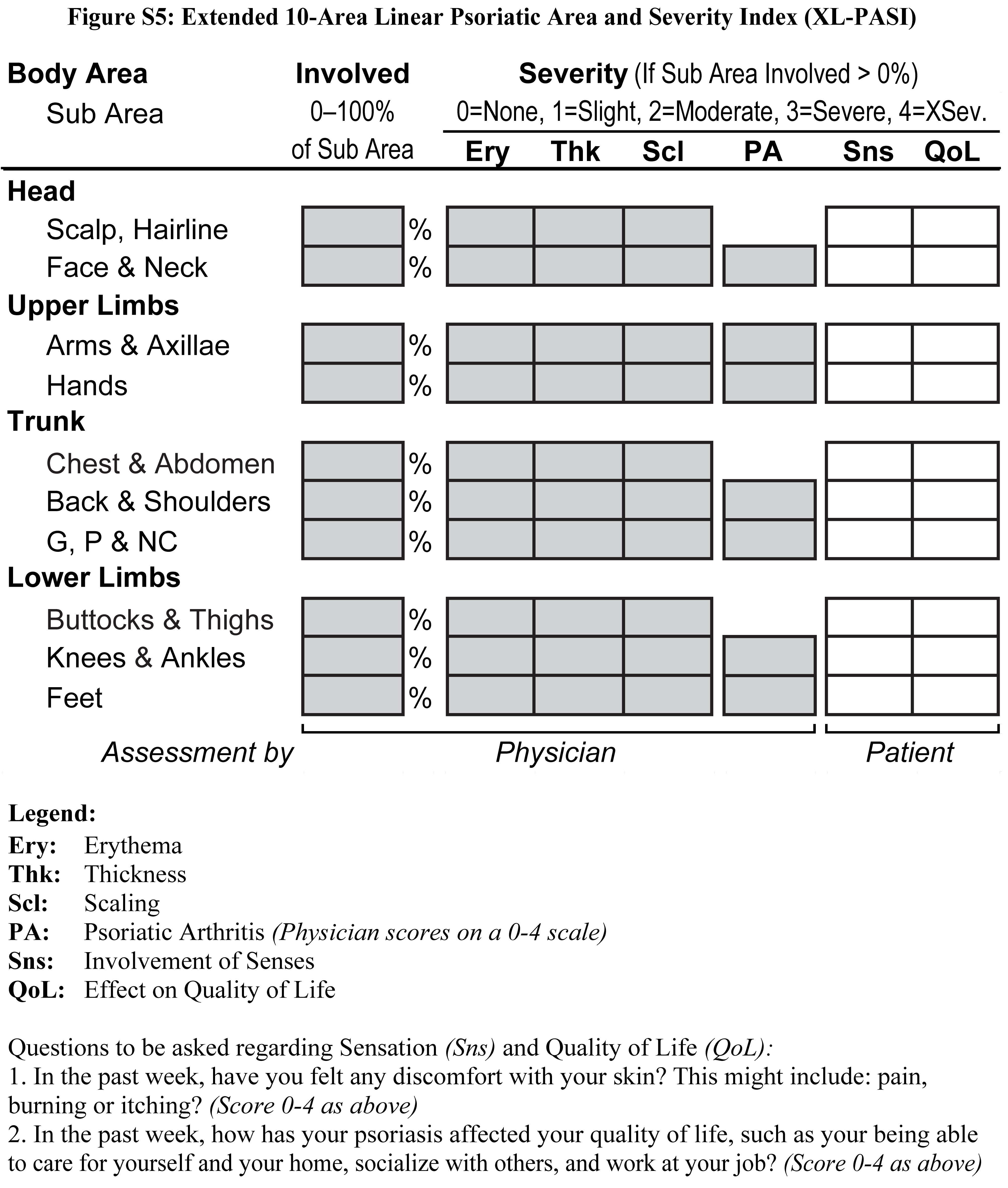

Supplement: Figure S5 — Extended 10-area Linear Psoriasis Area and Severity Index (XL-PASI) combines the PASI and PWESI scoring methods. This measure includes assessment of surface area involved, as well as dimensions for scaling, erythema, thickness and joint involvement for specific areas of psoriatic involvement. As with the PASI, severity indicators range from 0 (none) to 4 (extremely severe). As with the PWESI, body surface area is divided into ten (“X”) areas and each area is quantified. The scale of the XL-PASI ranges from 0 to 148. (TIF) [file pone.0020279.s005.tif]
